# Development of a modified Respiration Activity Monitoring System for accurate and highly resolved measurement of respiration activity in shake flask fermentations

**DOI:** 10.1186/1754-1611-6-11

**Published:** 2012-08-17

**Authors:** Sven Hansen, Ioanna Hariskos, Bettina Luchterhand, Jochen Büchs

**Affiliations:** 1 AVT. Biochemical Engineering, RWTH Aachen University, Worringerweg 1, Aachen, 52074, Germany

**Keywords:** Oxygen transfer rate, RAMOS, Shake flask, Bioprocess monitoring, Diauxic growth, Oxygen limitation

## Abstract

**Background:**

The Respiration Activity Monitoring System (RAMOS) is an established device to measure on-line the oxygen transfer rate (OTR), thereby, yielding relevant information about metabolic activities of microorganisms and cells during shake flask fermentations. For very fast-growing microbes, however, the RAMOS technique provides too few data points for the OTR. Thus, this current study presents a new model based evaluation method for generating much more data points to enhance the information content and the precision of OTR measurements.

**Results:**

In cultivations with *E.coli* BL21 pRSET eYFP-IL6, short diauxic and even triauxic metabolic activities were detected with much more detail compared to the conventional evaluation method. The decline of the OTR during the stop phases during oxygen limitations, which occur when the inlet and outlet valves of the RAMOS flask were closed for calibrating the oxygen sensor, were also detected. These declines reflected a reduced oxygen transfer due to the stop phases. In contrast to the conventional calculation method the new method was almost independent from the number of stop phases chosen in the experiments.

**Conclusions:**

This new model based evaluation method unveils new peaks of metabolic activity which otherwise would not have been resolved by the conventional RAMOS evaluation method. The new method yields substantially more OTR data points, thereby, enhancing the information content and the precision of the OTR measurements. Furthermore, oxygen limitations can be detected by a decrease of the OTR during the stop phases.

## Background

Shake flasks are widely used in fermentations for biotechnological research and industrial process development [1,2]. For gaining a better understanding and control of shake flask cultivations, various methods have been recently developed for online monitoring of process parameters.

Monitoring of pH-values in shake flasks has been realized both with standard autoclavable pH-probes that are immersed in the bulk liquid [3] and with fluorescent optodes fixed at the flask wall that allow optical measurement [4-6]. Moreover, dissolved oxygen tension (DOT) can in principle be measured in shake flasks by using either Clark-type electrodes [7-10] or optical sensors based on dynamic quenching of luminescence [11-16]. These non-invasive measurement methods have proven to be more reliable, since they do not alter the hydrodynamics of the culture system due to baffling effects [17,18].

As shown in numerous studies, almost all metabolic activities of aerobic microorganisms depend on the oxygen consumption of the culture [8,19-21]. Therefore, online measuring techniques are also useful for determining the gas transfer rates in shake flasks. The company BlueSens GmbH (Herten, Germany) has developed a system that measures the gas transfer rates in the headspace of shake flasks down to nominal flask volumes of 500 mL [22].

**Figure 1:**
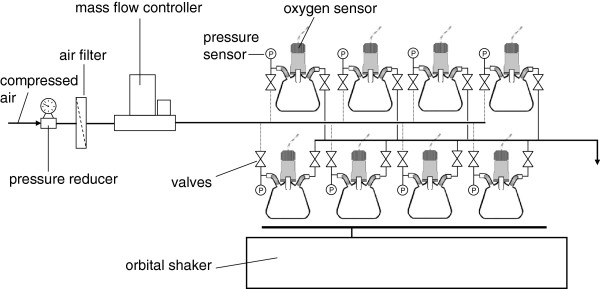
**Setup of a RAMOS device as introduced by Anderlei et al.**[23,24].

As an alternative method, the Respiration Activity Monitoring System (RAMOS) presented by Anderlei et al. [23,24], represents another non-invasive measurement technique which allows the online determination of oxygen transfer rate (OTR), carbon dioxide transfer rate (CTR) and respiratory quotient (RQ) in shake flasks down to 100 ml. Since its introduction, RAMOS has been used for multiple applications: e.g., for determining oxygen limitations in shake flask cultivations [18,25-30], screening of microorganisms [31-33], optimizing media [34-36], investigating secondary substrate limitations [37,38] and stress phenomena [39], process development and optimization [2,40-44] and for monitoring precultures for fermentations in stirred tank reactors [45,46]. The setup of a RAMOS device is illustrated in Figure 1. For determining OTR with RAMOS, a certain measuring cycle is regularly repeated and consists of a rinsing phase and a stop phase [23,24]. In the rinsing phase air is injected into the RAMOS flask to ensure a headspace gas concentration that equals that in a normal Erlenmeyer flask [24]. During the stop phase the air flow into the system is briefly interrupted, and then the OTR is calculated from the corresponding drop (linear slope) in the oxygen partial pressure as detected by an oxygen sensor. Since these measuring cycles are typically repeated every 30 min, a data density of merely two OTR measurement points per hour is usually achieved. 

The objective of this study is to present a new model based evaluation method for generating substantially more data points to enhance the information content and the precision of OTR measurements. First, the conventional way of generating OTR data is recalled. Then, the new evaluation method based on a complete oxygen headspace balance is developed. Microbial cultivations have been devised in order to evaluate the new method.

## Results and discussion

### Conventional evaluation method of the RAMOS device

In the conventional approach as proposed by Anderlei et al. [23,24], the OTR in RAMOS is calculated during the stop phase based on equation A.1. The oxygen sensor does not directly provide the oxygen partial pressure p_O2_ but a voltage signal U_O2_ that depends on it. Therefore, the expression $\frac{p_{O_{2},real}}{U_{O_{2},stop}}$ is introduced to convert this voltage signal into the oxygen partial pressure during the stop phase as described by the right part of equation A.1. Here, V_G_ and V_L_ are the volumes of the gas headspace and the liquid in the flask, respectively, and T is the temperature.

(1) $$OTR_{\mathrm{stop}}=\frac{\Delta p_{{o_{2}}_{\mathrm{stop}}}}{\Delta t}\cdot \frac{V_{G}}{V_{L}\cdot R\cdot T}=\frac{p_{o_{2},real}}{U_{o_{2},stop}}\cdot \frac{\Delta U_{o_{2}stop}}{\Delta t}\cdot \frac{V_{G}}{V_{L}\cdot R\cdot T}$$

**Figure 2:**
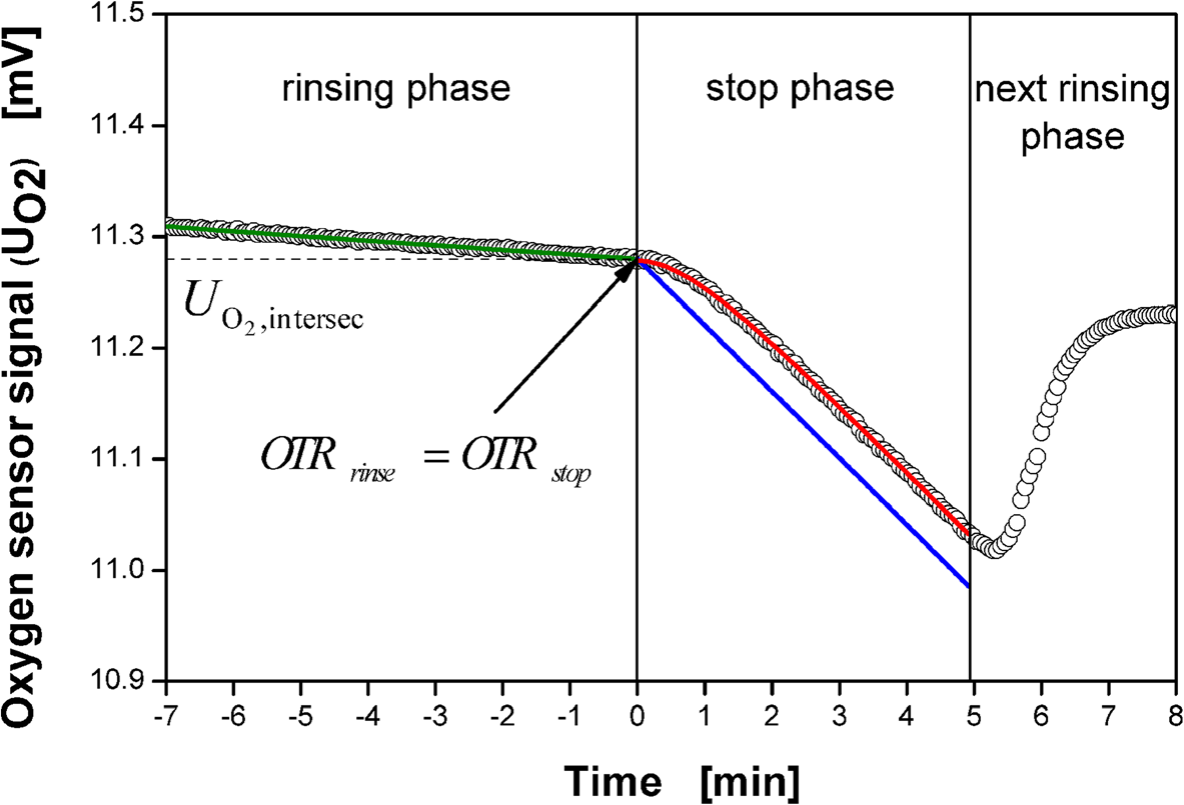
**Example of a calibration during a RAMOS measurement, with measured data from the oxygen sensor (*circle*), smoothing spline fit of the rinsing phase according to equation (**B.3**) (*green line*), optimized polynomial of the stop phase according to equation (**B.5**) used for solving equation (**B.4**) (*blue line*) and delayed signal of the stop phase according to the delay model described by equation (**B.6**) (*red line*.)**

To compensate for the signal drift, the sensors are calibrated by calculating the partial pressure of oxygen at the end of the rinsing phase $p_{O_{2},real}$ by applying the steady state gas composition of the headspace volume at 0 h. Figure 2 gives an example of the sensor signal during a stop phase. This leads to equation A.2 for calculating the OTR:

(2) $$OTR_{\mathrm{stop}}=\frac{\Delta {U_{O_{2}}}_{,stop}}{\Delta t}\cdot \frac{P_{O_{2},in}\cdot V_{G}\cdot V_{\mathrm{in}}}{U_{O_{2}}\cdot V_{L}\cdot T\cdot V_{\mathrm{in}}+P_{\mathrm{amb}}\cdot VG\cdot V_{m\cdot }\frac{\Delta U_{O_{2},stop}}{\Delta t}}$$

Here, p_O2,in_ is the oxygen partial pressure of the inlet flow, and ${\overset{\cdot }{\text{V}}}_{\mathrm{in}}$ is the volumetric flow into the flask during the rinsing phase, p_amb_ is the ambient pressure and V_m_ is the molar volume.

A calibration factor (K) can then be calculated by using equation (A.3).

(3) $$K=\frac{OTR_{\mathrm{stop}}}{\frac{\Delta U_{O_{2},stop}}{\Delta t}}\frac{V_{L}\cdot R\cdot T}{V_{G}}$$

**Figure 3:**
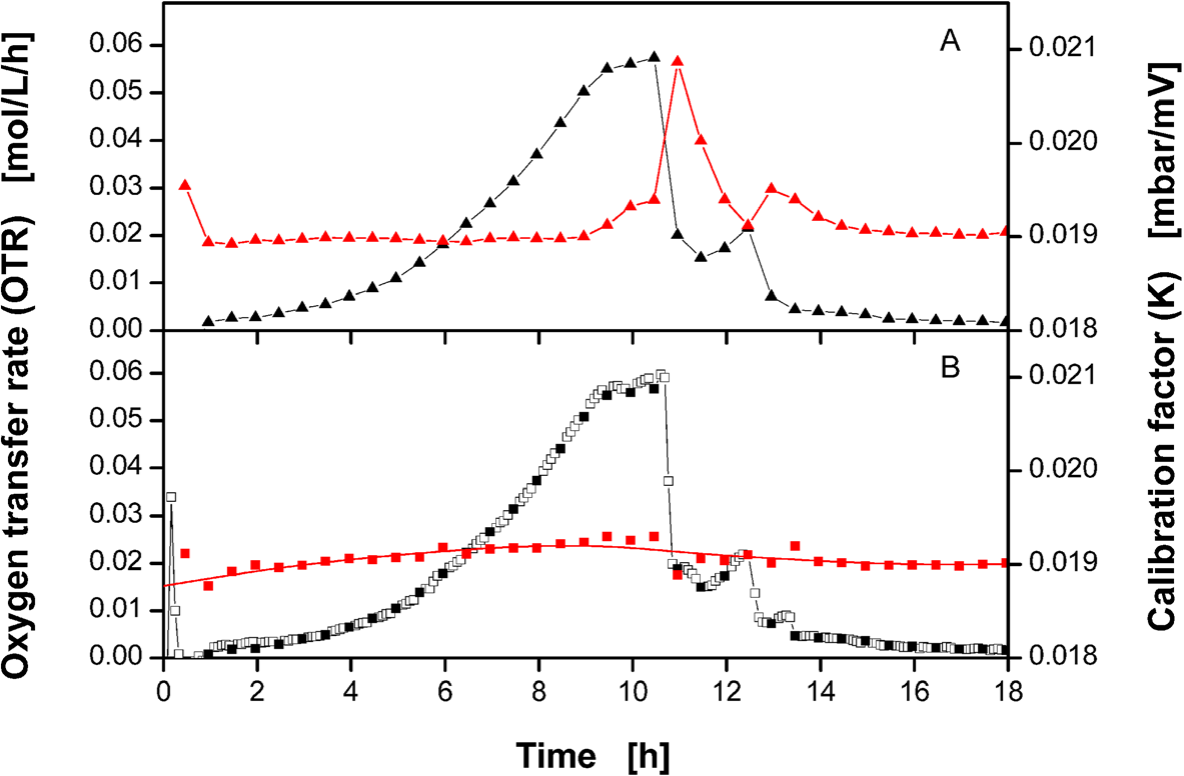
**Oxygen transfer rates (OTR) and calibration factors (K) of cultures of *E. coli *BL21 pRSET eYFP-IL6 in mineral medium measured with the RAMOS device by applying both the method of Anderlei et al.**[23,24]**and the newly developed evaluation method.A**: OTR calculated by applying the evaluation method of Anderlei et al. [23,24] using equation A.2 (*black triangle*) and the corresponding calibration factor K calculated with equation A.3 (*red triangle*); **B**: OTR calculated by applying the new evaluation method using equation (B.1) in the rinsing phase (*open square*), equation (B.4) in the stop phase (*black square*) and K, calculated as illustrated in Figure 2 and equation (B.7) (*red square*) with its smoothing spline fit (*red line*)**;** experimental conditions: Wilms-MOPS medium with 20 g/L glucose; temperature T = 30°C; filling volume V_L_ = 10 mL; shaking diameter d_0_ = 50 mm; shaking frequency n = 350 rpm; initial optical density OD_600_ = 0.2; initial pH = 7.5; rinsing phase t_rinse_ = 25 min; stop phase t_stop_ = 5 min; sensor lag time τ = 0.013 h.

To examine the conventional evaluation method presented by Anderlei et al. [23,24]*E.coli* BL21 pRSET eYFP-IL6 was cultivated in Wilms-MOPS-medium as described in “Materials and methods”. Figure 3A illustrates the OTRs calculated using equation (A.2). Here, the OTR curve consists of one measuring point every 30 min, because the OTR is only evaluated during the stop phases. This curve shows a typical diauxic growth with the first peak leveling off and forming a horizontal plateau between 9.5 h and 10.5 h after inoculation. At the end of this phase glucose is fully consumed under oxygen limitation [23,24]. At around 12.5 h after inoculation, a second peak appears indicating the consumption of acetate formed during the first phase of glucose consumption. This typical growth behavior has already been confirmed in other studies [6,47,48]. Therefore, the conventional evaluation method of RAMOS is able to measure the respiration activity of shake flask cultures and to detect phenomena as oxygen limitations and diauxic growth behavior, as long as these effects are lasting longer than the typical measuring cycle of 30 min. This condition is valid for many biological systems. However, a higher data density would be desirable when a highly resolved measurement is needed, e.g. for tracking metabolic activities that take place within much shorter periods. 

Also illustrated in Figure 3A is the calibration factor K as calculated by equation (A.3). This curve also shows two peaks which both occur right after the OTR peaks. These two peaks are totally unexpected, because the calibration factor K is assumed to be steady and independent from the OTR. Consequently, the assumption that the gas headspace volume at the end of the rinsing phase is in a steady state results in an inadequate calibration. Therefore, a calculation method should be developed that increases the data density of the OTR and includes a more accurate calibration.

## New model based evaluation method

For increasing the data density of the OTR measurement the data from the rinsing phase should also be utilized for the evaluation. Therefore, the complete oxygen balance of the headspace of a RAMOS flask is derived and resolved for the OTR as follows (equation (B.1)):

(4) $$OTR_{\mathrm{rinse}}=\frac{{\overset{\cdot }{V}}_{\mathrm{in}}\cdot P_{O2,in}-{\overset{\cdot }{V}}_{\mathrm{out}}\cdot K\cdot U_{O_{2},rinse}}{V_{L}\cdot R\cdot T}-K\cdot \frac{dU_{O_{2}.rinse}}{dt}\cdot \frac{V_{G}}{V_{L}\cdot R\cdot T}$$

${\overset{\cdot }{V}}_{\mathrm{in}}$ and ${\overset{\cdot }{V}}_{\mathrm{out}}$ describe the volumetric flows into and out of the flask, respectively, p_O2,in_ the oxygen partial pressure of the inlet flow, K is the calibration factor of the oxygen sensor, U_O2_ is the oxygen sensor signal, V_G_ and V_L_ are the volumes of the gas headspace and the liquid medium in the flask, respectively and T is the temperature.

The outlet flow ${\overset{\cdot }{V}}_{\mathrm{out}}$ is calculated from the slope of the pressure sensor signal of the headspace volume in the adjacent stop phases according to equation (B.2). This change in pressure during the stop phases occurs when the respiratory quotient (RQ) differs from 1. During the rinsing phases, when the valves are opened, this results in a change of the outlet flow. Equation (B.2) also takes into account the water vapor partial pressure that affects the outlet flow.

(5) $${\overset{\cdot }{V}}_{\mathrm{out}}={\overset{\cdot }{V}}_{\mathrm{in}}\frac{P_{\mathrm{amb}}}{P_{\mathrm{amb}}-P_{H2O}}+\frac{P_{amb,stop}}{dt}\cdot \frac{V_{G}}{P_{\mathrm{amb}}-P_{H2O}}$$

Here, p_amb_ describes the ambient pressure and p_H2O_ is the water vapor partial pressure in the gas headspace volume.

For determining the slope of the oxygen sensor signal $\frac{dU_{O_{2},rinse}}{dt}$, the sensor data of each rinsing phase are approximated with a smoothing spline function f that minimizes equation (B.3) according to the algorithm of de Boor [49]. This algorithm is implemented into the Curve Fitting Toolbox of MATLAB R2010b (The Math Works, MA, USA). The left part of equation (B.3) consists of a least square method and is responsible for a close approximation of the sensor signal. In contrast, the right part of the equation represents the second derivative of the fit and, therefore, is responsible for the smoothness required for calculating reliable derivations. For adjusting a good trade-off between these two terms the regularization parameter p is used. A regularization parameter of p = 0.999 is suitable to describe the sensor signal of the rinsing phases and is, therefore, used to perform the fitting

(6) $$p\sum_{j=1}^{n}{\left|U_{O_{2},rinse}\left(j\right)-f\left(t\left(j\right)\right)\right|}^{2}+\left(1-p\right)\int {\left|\frac{d^{2}f\left(t\right)}{dt^{2}}\right|}^{2}dt$$

p is the regularization parameter for the smoothing spline fitting and f describes the smoothing spline function.

For calculating the OTR in the stop phase, equation (B.1) simplifies to equation (B.4) since no air flow is applied to the flask. Therefore, ${\overset{\cdot }{V}}_{\mathrm{in}}$ and ${\overset{\cdot }{V}}_{\mathrm{out}}$ are zero and the left part of equation (B.1) vanishes.

(7) $$OTR_{\mathrm{stop}}=-K\cdot \frac{dU_{O_{2},stop}}{dt}\cdot \frac{V_{G}}{V_{L}\cdot R\cdot T}$$

The oxygen partial pressure in the stop phase is approximated with a second-order polynomial as described by equation (B.5). The reason for using a second-order polynomial equation is because it also considers a change of OTR during the stop phase and uses just one parameter more than a linear slope. As the stop phases are relatively short a more complicated equation resulting in a higher computational effort does not seem to be reasonable. Since the changes of the OTR are not restricted to the rinsing phase this method can be considered to be more accurate than using a linear slope. The first derivative of this polynomial is inserted into equation (B.4).

(8) $$U_{O_{2},stop}=at^{2}+bt+c$$

Due to the inertia of the oxygen sensor, there is a lag time for the sensor signal which does not allow a direct fit of equation (B.5). This lag time is compensated for by incorporating the first order delay model equation (B.6) into the fitting procedure. Thereby, the calculation of the coefficients a, b and c of the polynomial (B.5) is based on a least-square optimization procedure of these coefficients. Equation (B.6) is used to constantly calculate the corresponding sensor response U_O2,delay_ on equation (B.5) by varying the estimates of a, b and c. As soon as the deviation between the calculated sensor response U_O2,delay_ and the measured signal is small enough, the optimization stops and the course of the oxygen partial pressure can be calculated by using the corresponding estimates of the coefficients for the polynomial (B.5). Figure 2 also shows the optimized polynomial of the stop phase according to equation (B.5) and the delayed signal of the stop phase according to the delay model described by equation (B.6). For the oxygen sensors used in this work, a time constant τ of 0.013 h is used (see Materials and methods). This value best describes the dynamic behavior of the sensor.

(9) $$\frac{dU_{O_{2},delay}}{dt}=\frac{U_{O_{2},stop}-U_{O_{2},delay}}{\tau }$$

### Sensor calibration

For solving equations (B.1) and (B.4), the calibration factor K is required. A new calibration method has been developed which also considers the dynamic behavior of the gas headspace volume instead of assuming a steady state. The end of the rinsing phase as well as the start of the subsequent stop phase of a biological experiment is utilized in the new sensor calibration method. This point occurs at 0 h in Figure 2.

Figure 2 also shows a section of the sensor signal curve during a RAMOS measurement and the mathematical fits including both the rinsing phase and the stop phase. Within the rinsing phase which last up to the beginning of the stop phase at 0 min, the sensor signal is described by a smoothing spline function f according to equation (B.3). During the stop phase, from 0 min to 5 min, the sensor signal strongly decreases. However, due to the lag time at the beginning of this stop phase, the initial slope of the signal curve merely changes slowly. Figure 2, thus, shows both the polynomial described by equation (B.5) and the delayed signal described by equation (B.6) after the optimization procedure.

To calculate the calibration factor K, equations (B.1) and (B.4) are used. Whereas equation (B.1) describes the OTR during the rinsing phase and equation (B.4) the OTR during the stop phase, both equations can be equalized at the intersection of the curves of the rinsing phase and the stop phase as illustrated in Figure 2. This is justified, because a sudden change in the OTR or K due to a change in the gas flow can be excluded. By resolving equations (B.1) and (B.4) under the assumption of their equality (OTR_rinse_ = OTR_stop_), equation (B.7) is obtained to calculate K.

(10) $$\;K=\frac{-{\overset{\cdot }{V}}_{\mathrm{in}}\cdot P_{O_{2},in}}{\left(\frac{dU_{O_{2},stop,\mathrm{int}er\sec}}{dt}-\frac{dU_{O_{2},rinse,\mathrm{int}er\sec}}{dt}\right)\cdot V_{G}-{\overset{\cdot }{V}}_{\mathrm{out}}\cdot U_{O_{2},\mathrm{int}er\sec}}$$

Using the same experiment, Figure 3B depicts the OTR curve resulting from the new calculation method. Here, an OTR is calculated every 5 min. The OTR data from the stop phase are in good agreement with the OTR data calculated using the method of Anderlei et al. [23,24]. The OTR data from the rinsing phase mostly concur with that of the stop phase. Only during the phase of oxygen limitation, indicated by the horizontal plateau of the OTR data at ca. 9.5 – 10.5 h [23,24], the OTR in the stop phase is somewhat lower than that in the rinsing phase.

**Figure 4:**
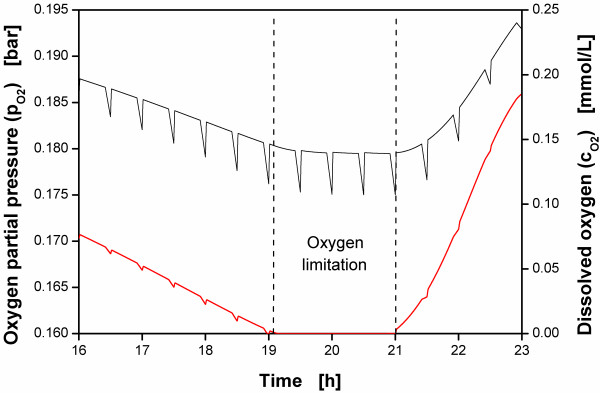
**Oxygen partial pressure of the gas headspace volume and dissolved oxygen during oxygen limitation.** Example of a RAMOS fermentation with oxygen partial pressure of the gas headspace volume (*black line*) and dissolved oxygen in the medium (*red line*) calculated with the model of equations (C.1) to (C.7) in the appendix.

To understand this phenomenon, Figure 4 is considered. The oxygen partial pressure of the gas headspace volume and the dissolved oxygen concentration in the medium of a simulation example are shown. The simulation is based on the model of equations (C.1) to (C.7) as shown in the appendix. This simulation example describes an oxygen limitation between 19 h and 21 h. The dissolved oxygen concentration c_O2_ was calculated by using the oxygen transfer equation (B.8). A constant mass transfer coefficient k_L_a was assumed.

(11) $$OTR=k_{L}a\cdot \left({c_{O2}}^{*}-c_{O2}\right)$$

Up to the beginning of the oxygen limitation at 19 h both the oxygen partial pressure of the headspace and the dissolved oxygen generally decrease due to increasing respiration activity. However, during the rinsing phases of this oxygen limitation between 19 h and 21 h the oxygen partial pressure of the headspace shows a horizontal plateau of ca. 0.18 bar whereas the dissolved oxygen has very low values which are close to the K_O2_ value of oxygen. Both the oxygen partial pressure of the headspace and the dissolved oxygen generally increase after the oxygen limitation when the respiration activity decreases.

Up to the oxygen limitation at 19 h, both, the oxygen partial pressure of the gas headspace volume and the dissolved oxygen strongly decrease during the stop phases as can be seen by the spikes in the signal. As both signals decrease the driving force of the oxygen transfer into the medium is not affected. However, during oxygen limitation between 19 h and 21 h the dissolved oxygen in the medium approaches very low values in the range of the K_M_ value for oxygen and cannot decrease further. Nevertheless, the oxygen partial pressure of the headspace decreases, resulting in a lower driving force for the oxygen transfer into the medium and, consequently, in a slightly lower OTR during the stop phases, as depicted for 9.5 h–10.5 h in Figure 3B.

In Figure 3B also the calibration factor K as calculated with equation (B.7) is shown. In contrast to the steady state calibration using equation (A.3) as shown in Figure 3A, the calibration factor K calculated with equation (B.7) has a steady course as expected and does not show any peaks correlated to the OTR. However, the data for K show some fluctuations which are due to the lower accuracy of data fitting at higher sensor dynamics. Therefore, the data for K are smoothed by using a smoothing spline with a regularization parameter of p = 0.1. This value has been found to be a good trade-off between smoothing of K and revealing the effect of sensor drift.

### Case studies

#### Recombinant *E. coli* fermentation in mineral medium

**Figure 5:**
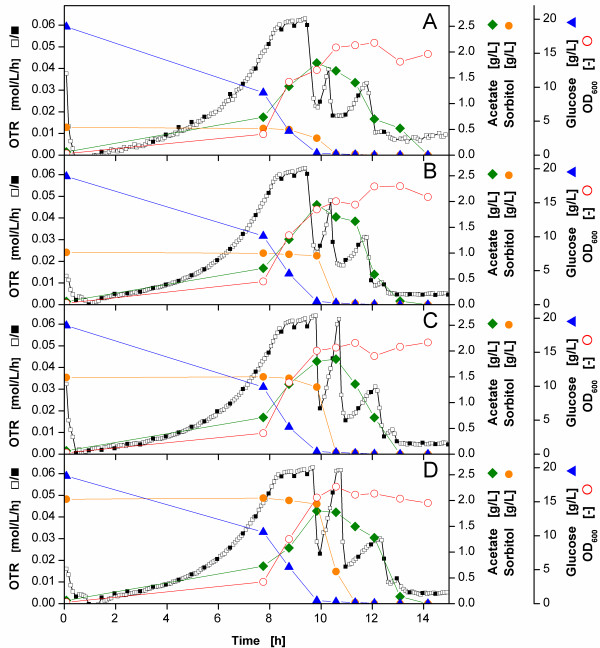
**Oxygen transfer rates (OTR) of *E. coli *BL21 pRSET eYFP-IL6 in mineral medium with different initial sorbitol concentrations.A**: 0.5 g/L sorbitol; **B**: 1 g/L sorbitol; **C**: 1,5 g/L sorbitol; **D**: 2 g/L sorbitol; RAMOS data from the rinsing phase calculated with equation (B.1) (*open square*) and from the stop phase calculated with equation (B.4) (*black square*); glucose concentration (*blue triangle*); sorbitol concentration (*orange circle*); acetate concentration (*green diamond*); optical density (OD_600_) at 600 nm (*red open circle*); experimental conditions: Wilms-MOPS medium with 20 g/L glucose and different sorbitol concentrations; temperature T = 30°C; filling volume V_L_ = 10 mL; shaking diameter d_0_ = 50 mm; shaking frequency n = 350 rpm; initial optical density OD_600_ = 0.2; initial pH = 7.5; rinsing phase t_rinse_ = 25 min; stop phase t_stop_ = 5 min; sensor lag time τ = 0.013 h.

To verify the new evaluation method, *E.coli* BL21 pRSET eYFP-IL6 was cultivated using a RAMOS device with Wilms-MOPS-medium containing glucose and sorbitol as carbon sources. Four shake flasks were examined, each having a different initial sorbitol concentration. Figure 5 shows the results. According to Monod [50], this cultivation leads to a diauxic growth where first glucose is consumed followed by a consumption of sorbitol. In all four cases, the OTR curves show an exponential growth phase during the 8 h after inoculation. Afterwards, the OTR curves form a horizontal plateau for up to 9.75 h after inoculation which indicates an oxygen limitation during this period of time [23,24]. This oxygen limitation becomes more apparent when considering that the OTR of the stop phases are slightly lower during this period compared to the OTR of the rinsing phases. This difference is attributed to a reduced driving force of the oxygen transfer during the stop phases at oxygen limitation as described above. The oxygen concentration in the medium approaches very low values in the range of the K_O2_ value for oxygen, whereas the oxygen partial pressure of the headspace is still decreasing. Up to 9.75 h after inoculation when the oxygen limitation ends and the OTR decreases, glucose is completely consumed. Before glucose is depleted, the sorbitol concentrations remain constant and acetate is formed as an overflow metabolite of *E. coli* fermentation [6,47,48]. Furthermore, the optical density increases indicating biomass formation. 

After 9.75 h, when the glucose is fully exhausted, sorbitol is consumed as can be seen by a decline in the sorbitol concentration. This sorbitol consumption is also observed in the OTR curve via a peak beginning at 10 h. The higher the initial concentration of sorbitol, the more pronounced the peak of the OTR curve is (from Figure 5A to 5D). Whereas the second OTR peak reaches a maximum of 0.039 mol/L/h at an initial sorbitol concentration of 0.5 g/L (Figure 5A), it attains a maximum of 0.048 mol/L/h at an initial sorbitol concentration of 1 g/L (Figure 5B) and a limiting value of 0.063 mol/L/h at an initial sorbitol concentration of 1.5 g/L (Figure 5C). At an initial sorbitol concentration of 2 g/L (Figure 5D), the OTR peak also reaches limiting values at about 0.062 mol/L/h, but is much wider. Thus, due to the higher data density, the new evaluation method unveils the real size of these peaks.

When sorbitol is depleted, the OTR curve again decreases before rising up to form a third peak which infers that acetate is now being consumed. This third peak has the same size for all four initial sorbitol concentrations, because the acetate is formed during the phase of glucose consumption and not during the sorbitol consumption. When acetate is consumed biomass is not produced anymore. Consequently, in the historical work of Monod [50], who measured biomass, only diauxic growth was observed and not a triauxic metabolic activity, as observed with the newly proposed calculation method of RAMOS data.

**Figure 6:**
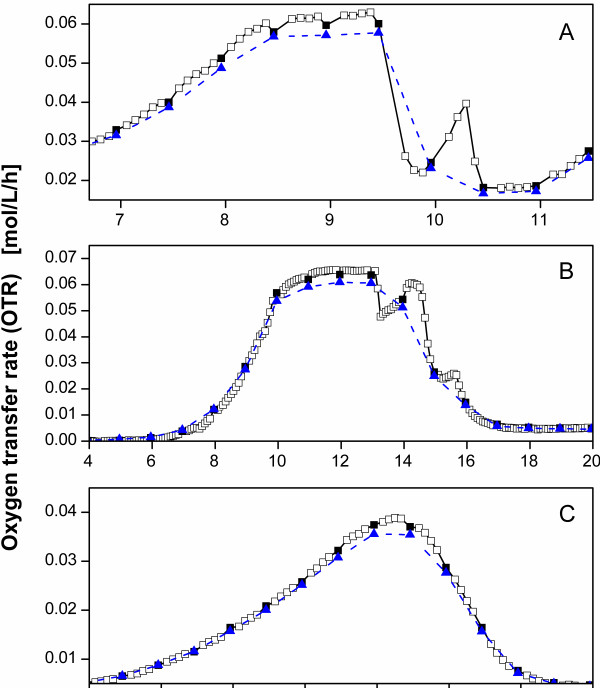
**Comparison of the method of Anderlei et al.**[23,24]**using equation (A.2) (*blue triangle*) and the new evaluation method using equation (B.1) in the rinsing phase (*open square*) and equation (B.4) in the stop phase (*black square*).A**: Enlarged section of the OTR curve of Figure 5A; **B**: *E. coli* BL21 pRSET eYFP-IL6 in TB medium; temperature T = 30°C; filling volume V_L_ = 10 mL; shaking diameter d_0_ = 50 mm; shaking frequency n = 300 rpm; inoculated with 0.2 ml cryo culture; rinsing phase t_rinse_ = 55 min; stop phase t_stop_ = 5 min; high flow phase t_high_ = 0.9 min; **C:***Gluconobacter oxydans* 621 H wildtype in complex medium with 40 g/L mannitol and 0.5 g/L glycerol; temperature T = 30°C; filling volume V_L_ = 10 mL; shaking diameter d_0_ = 50 mm; shaking frequency n = 350 rpm; initial optical density OD_600_ = 0.1; rinsing phase t_rinse_ = 25 min; stop phase t_stop_ = 5 min; a sensor lag time of τ = 0.013 h is used for all experiments.

To show the strengths of the new evaluation method, three fermentation examples were considered where the OTR data of both methods are shown. Figure 6A illustrates an enlarged section of the OTR curve of Figure 5A. This OTR curve indicates an oxygen limitation between 8.5 h and 9.5 h as the OTR data points of the rinsing phases form a horizontal plateau during this time [23,24]. Subsequently, the OTR drops sharply, indicating a reduced respiration activity until 11 h after inoculation. The OTR curve calculated with the new method generates a measurement point every 5 min, hence, offers a very high data density. Therefore, a second peak between 10 h and 10.5 h could be detected which is due to the consumption of sorbitol as could be shown in Figure 5A. This shows that the newly proposed method is very useful in displaying short-term effects of the OTR. 

The OTRs calculated with the new evaluation method in the stop phase are slightly higher than those calculated with the method of Anderlei et al. [23,24]. This deviation is caused by the consideration of the sensor lag time in the new calculation method (equation (B.6)).

Using the new method, it is clearly observed that during the oxygen limitation between 8.5 h and 9.5 h the data points in the stop phase (filled squares) are not in line with the data points from the rinsing phase (open squares), in contrast to the rest of the fermentation. As discussed previously, this difference is due to a reduced driving force of the oxygen transfer during the stop phases of an oxygen limitation. The oxygen concentration in the medium approaches very low values, whereas the partial pressure of oxygen in the gas headspace volume still decreases. This effect does not occur in usual Erlenmeyer flasks, where the diffusive mass transfer through the cotton plug is not interrupted. Consequently, the OTR of the stop phase, gives a slightly different OTR than appearing in a typical Erlenmeyer flask.

#### Recombinant *E. coli* fermentation in complex medium

Figure 6B illustrates the OTR curves of a RAMOS cultivation of *E.coli* BL21 pRSET eYFP-IL6 in TB-medium. Instead of the typically chosen value of 25 min, the rinsing phase was set to a duration of 55 min. However, the data density of the OTR curve calculated with the new evaluation method is not affected by the reduced number of stop phases because the data of the rinsing phase are also considered. After the lag phase at 7 hours the OTR shows an exponential growth up to 10.5 h leading directly into a horizontal plateau which can be considered as an oxygen limitation [23,24]. Afterwards, even two very distinct peaks can be seen between 13 h and 16 h before the OTR drops down to a level of 0.005 mol/L/h at ca. 17 h. This clearly shows that the new method is also independent from the number of stop phases selected in the experiments. This characteristic is advantageous in applications where short-term effects are expected but a high number of stop phases is not desired, e.g. for preventing further oxygen limitations due to frequent interruption of the air flow.

#### *Gluconobacter oxydans* fermentation in complex medium

Figure 6C illustrates the OTR curves of an RAMOS cultivation of *Gluconobacter oxydans* 621 H wild type in complex medium with 40 g/L mannitol. A rinsing phase of 25 min was selected. The OTR curve calculated with the method of Anderlei et al. [23,24] indicates an exponential growth of the culture up to 7 h of cultivation when an OTR of 0.035 mol/L/h is achieved. The next measuring point at 7.5 h also indicates an OTR of 0.035 mol/L/h before the OTR decreases again. Consequently, the time between 7 h and 7.5 h could be interpreted as a short period of oxygen limitation. Even though the OTR curve calculated with the new method basically shows the same course, the time span between 7 h and 7.5 h shows considerably more data points, and more importantly, the OTR curve with the new calculation method does not show an oxygen limitation between the two stop phases.

## Conclusions

The newly proposed evaluation method yields substantially more OTR data points than the conventional method by Anderlei et al. [23,24]. This new evaluation method unveils additional peaks of metabolic activity which otherwise would remain undetected by the former method. Consequently, this new technique is a sophisticated means to generate more detailed information about metabolic activities of any kind of microorganisms and cells during shake flask cultivations. Additionally, possible oxygen limitations can be detected by a decrease of the OTR during the stop phases of the RAMOS measurement.

## Materials and methods

### Organisms

*E.coli* BL21 pRSET eYFP-IL6 was maintained at −80°C in Lysogeny broth (LB) medium with 100 μg/mL ampicillin. Stock solutions contained 200 g/L glycerol. *Gluconobacter oxydans* 621 H wild type was maintained in its cultivation medium (see below) including 80 g/L mannitol at −80°C. Stock solutions contained 150 g/L glycerol.

### Media

LB medium for maintaining *E.coli* consists of: 5 g/L yeast extract (powdered, Roth, Karlsruhe, Germany), 10 g/L tryptone (pancreatic digest of casein, Roth, Karlsruhe, Germany) and 10 g/L NaCl. Terrific broth (TB) medium was used for cultivating both *E.coli* precultures and main cultures. The medium consists of: 5 g/L glycerol, 12 g/L tryptone (pancreatic digest of casein, Roth, Karlsruhe, Germany), 24 g/L yeast extract (powdered, Roth, Karlsruhe, Germany), 12.54 g/L K_2_HPO_4_ and 2.31 g/L KH_2_PO_4_. Additionally, 0.1 g/L ampicillin was added.

Main cultures of *E.coli* in mineral medium were cultivated in modified Wilms & Reuss synthetic medium (henceforth referred to as Wilms-MOPS medium) [6,51]. This medium consists of: 20 g/L glucose, 5 g/L (NH_4_)_2_SO_4_, 0.5 g/L NH_4_Cl, 3 g/L K_2_HPO_4_, 2 g/L Na_2_SO_4_, 0.5 g/L MgSO_4_·7H_2_O, 41.85 g/L (0.2 M) 3-(N-morpholino)-propanesulfonic acid (MOPS), 0.01 g/L thiamine hydrochloride, 1 mL/L trace element solution (0.54 g/L ZnSO_4_·7H_2_O, 0.48 g/L CuSO_4_·5H_2_O, 0.3 g/L MnSO_4_·H_2_O, 0.54 g/L CoCl_2_·6H_2_O, 41.76 g/L FeCl_3_·6H_2_O, 1.98 g/L CaCl_2_·2H_2_O, 33.39 g/L Na_2_EDTA (Titriplex III)) and concentrations of sorbitol ranging from 0 – 2 g/L. Additionally, 0.1 g/L ampicillin was added.

*Gluconobacter oxydans* was cultivated in a complex medium consisting of: 5 g/L yeast extract (powdered, Roth, Karlsruhe, Germany), 1 g/L K_2_HPO_4_, 1 g/L (NH_4_)_2_SO_4_, 0.5 g/L glycerol, 2.5 g/L MgSO_4_·7H_2_O and 40 g/L mannitol. The pH-value was adjusted to 6 with 1 M KOH.

### Cultivation conditions

*E. coli* precultures and main cultures were cultivated in 250 ml shake flasks at a temperature of T = 30°C with a shaking diameter of d_0_ = 50 mm and a shaking frequency of n = 350 rpm (shaking machine LS-W, Kuehner AG, Birsfelden, Switzerland). For *E. coli* precultures, 20 ml of TB medium was inoculated with 200 μl of stock solution. Main cultures of *E.coli* in 10 mL Wilms-MOPS medium were inoculated with preculture broth from the exponential growth phase, resulting in an optical density (OD) of 0.2.

The main culture of *E.coli* in TB medium was cultivated in 250 ml shake flasks with a filling volume of V_L_ = 10 mL, a shaking diameter of d_0_ = 50 mm, a shaking frequency of n = 300 rpm, and at a temperature of T = 30°C. This culture was inoculated with 200 μl of stock solution.

The preculture and the main culture of *Gluconobacter oxydans* were cultivated in 250 ml shake flasks with a filling volume of V_L_ = 10 mL, a shaking diameter of d_0_ = 50 mm, a shaking frequency of n = 350 rpm, and at a temperature of T = 30°C. The preculture was inoculated with 500 μL of stock solution. The main culture was inoculated with preculture broth from the exponential growth phase resulting in an OD of 0.1.

### Respiration Activity Monitoring System (RAMOS)

The respiration activity was measured in modified 250 mL Erlenmeyer flasks using a self-made RAMOS device as introduced by Anderlei et al. [23,24]. The setup of the RAMOS device is shown in Figure 1. The 8 RAMOS flasks in parallel were supplied with air using a mass flow controller (type 5850 TR, Brooks, Hatfield, PA, USA). The air flow through the flasks is controlled by using valves at the inlet and the outlet of each RAMOS flask. For the rinsing phase the RAMOS flasks were flushed for 25 min with air at a flow rate of (low flow), which corresponds to an aeration rate of 1 vvm at a filling volume of V_L_ = 10 mL. The rinsing phase was followed by a 5 min stop phase (t_stop_) with no air flow through the flasks. To compensate for the drop in oxygen partial pressure during the stop phase, a higher air flow of ${\overset{\cdot }{V}}_{in,high}=3.6\;L_{N}/\text{h}$ (high flow) was applied during the first 0.9 min of the rinsing phase. This measuring cycle is regularly repeated. The oxygen partial pressure of the headspace in a RAMOS flask was measured with a MAX250 oxygen sensor from Maxtec (Salt Lake City, Utah, USA). The difference between the headspace pressure and ambient pressure was detected with a 26PCA pressure sensor from Honeywell (Morristown, NJ, USA). The RAMOS flasks ensure the same hydrodynamic conditions and headspace gas concentrations as are found in regular Erlenmeyer flasks with cotton plugs [24]. Commercial versions of the RAMOS device are available from Kuehner AG, Birsfelden, Switzerland and Hitech Zang, Herzogenrath, Germany.

The sensor lag time τ has been determined in step change experiments. Beginning at a steady state of air in the gas headspace volume, oxygen depleted air was flushed into a RAMOS flask under well-defined conditions ( ${\overset{\cdot }{V}}_{\mathrm{in}}$ = 0.54 mL/min, p_O2,in_ = 0.197 bar, V_L_ = 10 mL, n = 300 rpm). By calculating the real partial pressure of oxygen in the gas headspace of the flask and then optimizing the sensor lag time τ, the model described by equation (B. 6) was fitted to the oxygen partial pressure signal measured by the sensor. This leads to a sensor lag time of τ = 0.013 h (data not shown).

### Parallel shake flask cultivations

Samples were taken from *E. coli* cultivations in Wilms-MOPS medium in Erlenmeyer flasks in parallel to the RAMOS experiments and cultivated under the same conditions as used in the RAMOS experiments. The OD of the samples at 600 nm was determined with a Thermo Scientific Genesys 20 spectrophotometer (Waltham, MA, USA).

For determining glucose, sorbitol and acetate, the samples were centrifuged for 5 min at 14000 rpm with a Sigma 1–15 Microfuge (Osterode am Harz, Germany) and the supernatants were used for the analysis. Concentrations of glucose, sorbitol and acetate in the respective supernatants were determined using a Dionex HPLC (Dionex, Sunnyvale, USA) with an Organic Acid-Resin 300 × 8 mm (CSChromatographie, Langerwehe, Germany) and a Skodex RI-71 detector. Sulfuric acid in a concentration of 5 mM was used as solvent at a flow rate of 0.6 ml/min and a temperature of 60°C.

### Software

OTRs, the calibration factor (K) and simulation examples were calculated with MATLAB R2010b (The Math Works, MA, USA). The mathematical optimization problems were solved using the trust-region-reflective algorithm. Ordinary differential equations where solved using the trapezoidal rule.

## Appendix

Model used for calculating the data of Figure 4:Biomass equation:

(12) $$\frac{dX}{dt}=\mu \cdot X$$

Substrate equation:

(13) $$\frac{dS}{dt}=-\frac{1}{Y_{\mathrm{XS}}}\cdot \mu \cdot X$$

Dissolved oxygen equation:

(14) $$\frac{dc_{O2}}{dt}=-\frac{1}{Y_{XO2}}\cdot \mu \cdot X+OTR$$

Oxygen partial pressure equation:

(15) $$\frac{dp_{02}}{dt}=\frac{P_{02,in}\cdot {\overset{\cdot }{V}}_{\mathrm{in}}-{\overset{\cdot }{V}}_{\mathrm{out}}\cdot p_{o2}}{V_{G}}-OTR\cdot \frac{V_{L}\cdot R\cdot T}{V_{G}}$$

Growth rate:

(16) $$\mu =\mu_{\max}\cdot \frac{S}{S+K_{s}}\frac{c_{O2}}{c_{O2}+K_{O2}}$$

Oxygen transfer rate:

(17) $$OTR=k_{L}a\cdot \left({c_{O2}}^{*}-c_{O2}\right)$$

Dissolved oxygen at the gas–liquid interface:

(18) $${c_{O2}}^{*}=L_{O2}\cdot p_{O2}$$

With: Oxygen solubility L_O2_ = 0.0011 mol/L/bar, mass transfer coefficient k_L_a = 0.082 1/s, volumetric flow into the flask ${\overset{\cdot }{V}}_{\mathrm{in}}$ = 0.67 L/h, volumetric flow out of the flask ${\overset{\cdot }{V}}_{\mathrm{out}}$ = 0.7 L/h, maximal growth rate μ_max_ = 0.26 1/h, half velocity constant of the substrate K_S_ = 4 g/L, half velocity constant of oxygen K_O2_ = 8 ·10^-10^ mol/L, oxygen partial pressure of inlet p_O2,in_ = 0.2095 bar, yield coefficient of the substrate Y_XS_ = 0.5 g/g, liquid volume V_L_ = 10 mL, gas volume V_G_ = 270 mL, gas constant R = 0.0831 L*bar/mol/K, yield coefficient of oxygen Y_XO2_ = 41.6 g/mol, temperature T = 303.15 K, initial conditions: biomass concentration X_o_ = 0.5 g/L, substrate concentration S_0_ = 45 g/L, oxygen concentration in the liquid c_O2,0_ = 0.22 mmol/L, oxygen partial pressure p_O2,0_ = 0.2006 bar.

## Nomenclature

a, b, c: Polynomial coefficients; c_O2_: Dissolved oxygen in the liquid (mmol/L); c_O2,0_: Initial dissolved oxygen in the liquid (mmol/L); c_O2_^*^: Dissolved oxygen at the gas–liquid interface (mmol/L); f: Smoothing spline; k_L_a: Mass transfer coefficient (1/s); K: Calibration factor (bar/mV); K_O2_: half velocity constant for oxygen (mol/L); K_S_: Half velocity constant for the substrate (g/L); L_O2_: Oxygen solubility (mol/L/bar); OTR: Oxygen transfer rate (mol/L/h); p: Regularization parameter (-); p_amb_: Ambient pressure (bar); p_H2O_: Water vapor partial pressure in the headspace (bar); p_O2_: Oxygen partial pressure in the headspace (bar); p_O2,0_: Initial oxygen partial pressure in the headspace (bar); p_O2,in_: Oxygen partial pressure of inlet flow (bar); p_O2,real_: Steady state oxygen partial pressure (bar); R: Gas constant (bar L/mol/K); RQ: Respiratory quotient (-); S: Substrate concentration (g/L); S_0_: Initial substrate concentration (g/L); T: Temperature (K); U_O2_: Oxygen sensor signal (mol/L/h); V_G_: Gas volume (L); V_L_: Liquid volume (L); V_m_: Molar volume (L/mol); ${\overset{\cdot }{V}}_{\mathrm{in}}$: Volumetric flow into the flask (L/h); ${\overset{\cdot }{V}}_{\mathrm{out}}$: Volumetric flow out of the flask (L/h); X: Biomass concentration (g/L); X_0_: Initial biomass concentration (g/L); Y_XO2_: Yield coefficient for oxygen (g/mol); Y_XS_: Yield coefficient for the substrate (g/g); μ: Growth rate (1/s); μ_max_: Maximal growth rate (1/s); t: Lag time of oxygen sensor (h).

## Competing interests

The authors declare that they have no competing interests.

## Authors’ contributions

SH drafted the manuscript and performed the calculations. SH, IH and BL performed the experiments. Having conceived the study, JB helped to design, coordinate and write the manuscript. All authors read and approved the final manuscript.
